# Supplementary material for: Causal Relationship between Aging and Anorexia Nervosa: A White-Matter-Microstructure-Mediated Mendelian Randomization Analysis
Source: Biomedicines. 2024 Aug 16;12(8):1874. doi: 10.3390/biomedicines12081874 (PMC11351342; doi:10.3390/biomedicines12081874)
Supplement: Supplementary file 1 [file biomedicines-12-01874-s001.zip › Supplementary Figure/Figure S4/Singe SNP plot of MR analysis for TL to ubm-b-1474.pdf]

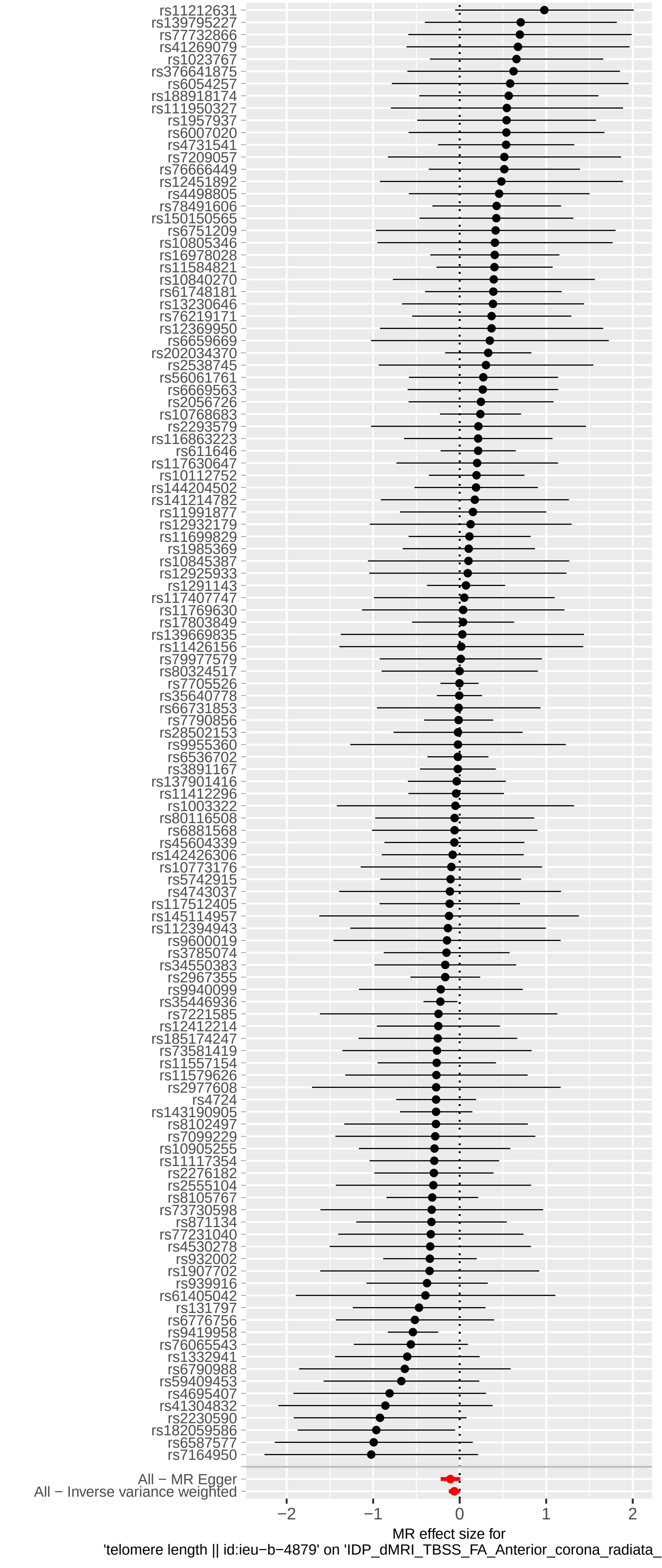
rs11212631
rs139795227
rs77732866
rs41269079
rs1023767
rs376641875
rs6054257
rs188918174
rs111950327
rs1957937
rs6007020
rs4731541
rs7209057
rs76666449
rs12451892
rs4498805
rs78491606
rs150150565
rs6751209
rs10805346
rs16978028
rs11584821
rs10840270
rs61748181
rs13230646
rs76219171
rs12369950
rs6659669
rs202034370
rs2538745
rs56061761
rs6669563
rs2056726
rs10768683
rs2293579
rs116863223
rs611646
rs117630647
rs10112752
rs144204502
rs141214782
rs11991877
rs12932179
rs11699829
rs1985369
rs10845387
rs12925933
rs1291143
rs117407747
rs11769630
rs17803849
rs139669835
rs11426156
rs79977579
rs80324517
rs7705526
rs35640778
rs66731853
rs7790856
rs28502153
rs9955360
rs6536702
rs3891167
rs137901416
rs11412296
rs1003322
rs80116508
rs6881568
rs45604339
rs142426306
rs10773176
rs5742915
rs4743037
rs117512405
rs145114957
rs112394943
rs9600019
rs3785074
rs34550383
rs2967355
rs9940099
rs35446936
rs7221585
rs12412214
rs185174247
rs73581419
rs11557154
rs11579626
rs2977608
rs4724
rs143190905
rs8102497
rs7099229
rs10905255
rs11117354
rs2276182
rs2555104
rs8105767
rs73730598
rs871134
rs77231040
rs4530278
rs932002
rs1907702
rs939916
rs61405042
rs131797
rs6776756
rs9419958
rs76065543
rs1332941
rs6790988
rs59409453
rs4695407
rs41304832
rs2230590
rs182059586
rs6587577
rs7164950
All − MR Egger
All − Inverse variance weighted
−2
−1
0
1
2
MR effect size for
'telomere length || id:ieu−b−4879' on 'IDP_dMRI_TBSS_FA_Anterior_corona_radiata_
